# The human ACE-2 receptor binding domain of SARS-CoV-2 express on the viral surface of the Newcastle disease virus as a non-replicating viral vector vaccine candidate

**DOI:** 10.1371/journal.pone.0263684

**Published:** 2022-02-08

**Authors:** Bo-Kyoung Jung, Yong Hee An, Jin-Ju Jang, Joo Hee Jeon, Sung Hoon Jang, Hyun Jang

**Affiliations:** 1 Libentech Co. LTD, Daejeon, Republic of Korea; 2 Department of Biological Sciences, College of Natural Sciences, Inha University, Incheon, Republic of Korea; Instituto Butantan, BRAZIL

## Abstract

Since the SARS-CoV-2 infection was identified in December 2019, SARS-CoV-2 infection has rapidly spread worldwide and has become a significant pandemic disease. In addition, human death and serious health problem caused by SARS-CoV-2 infection, the socio-economic impact has been very serious. Here, we describe the development of the viral vector vaccine, which is the receptor-binding domain (RBD) of SARS-CoV-2 expressed on the surface of Newcastle disease virus (LVP-K1-RBD19). The RBD protein concentrations on the viral surface were measured by the sandwich ELISA method. 10^6.7^ TCID_50_/ml of LVP-K1-RBD19 has a 0.17 μg of RBD protein. Optical density (OD) values of mouse sera inoculated with 10 μg of RBD protein expressed on the surface of LVP-K1-RBD19 generated 1.78-fold higher RBD-specific antibody titers than mice inoculated with 10 μg RBD protein with alum at 28 dpi. Moreover, mice inoculated with 10 μg of RBD protein expressed on the surface of LVP-K1-RBD19 virus showed more than 80% neutralization at 1:256 against the SARS-CoV-2 pseudovirus. These results demonstrated that inactivated LVP-K1-RBD19 virus produces neutralizing antibodies against SARS-CoV-2 in a short period and could be elect protective immunity in humans and LVP-K1-RBD19 will be a good candidate for the COVID-19 vaccine.

## Introduction

Pneumonia like severe respiratory symptom patients infected with severe acute respiratory syndrome coronavirus 2 (SARS-CoV-2; COVID-19) was first reported in Wuhan, Hunan province, China in December 2019 [1–3]. Patients with pneumonia symptoms occurred mainly from visitors to the seafood market in Wuhan City, the virus was isolated from the patient by a scientist in Wuhan, and genetic analysis revealed that it is a novel coronavirus, but this virus has a different genome sequence from SARS-CoV and MERS-CoV [4, 5]. February 1, 2020, 11,821 patients having severe pneumonia as symptoms reported in China and 132 patients confirmed by new emerging virus and this virus infection reported in 23 countries including China [6]. The virus was isolated from the patient, named SARS-CoV-2 and transmitted rapidly around the world. Currently, more than one hundred million people are infected per day and more than two million people were died by virus infection. In Korea, on January 19, 2020, the first SARS-CoV-2 infected patient came out, and currently, more than 87,000 people were infected and 1,562 people were died by SARS-CoV-2 infection [7]. The major symptom of the SARS-CoV-2 infection are dry cough, fatigue, and loss of body aches, headaches, diarrhea, sore throat, loss of taste or smell, but symptoms variable from person to person, and even some people are doing not have any specific symptoms after viral infection [8, 9]. Virus transmission is very fast and the fatality rate is 1.36% in Korea and 3.0% in the world, but the fatal rate slowly decreases compares with the early starting virus transmission stage. Now the virus is transmitted to 221 countries every day 250,000 through 500,000 people are infected every day and 10,000 people have died. Since then 1918 Spanish flu, SARS-CoV-2 as a pandemic disease affect most seriously affect human health and the economy.

The SARS-CoV-2 virus is a beta-corona virus belong to *coronaviridae* [6, 10]. The SARS-CoV-2 virus has a single strand RNA genome ant it sizes up to 30,473 base pairs [11, 12]. The genome composes a total of 10 nonstructural and structural genes, ORF1a to ORF10. Among the genes, S gene coding the spike protein, which is structural gene binding ACE2 receptor of the Human host cell [13, 14]. At the beginning status of SARS-CoV-2 transmission, Genotype A of the SARS-CoV-2 virus was isolated from patients after that this type was prevalent in North America and Europe [15–17]. B type of the SARS-CoV-2 prevalent in East Asia and C type prevalent in Europe and some Asian countries including South Korea [15–17]. Genetic different types of the SARS-CoV-2 virus identified the same serotype, but new genetically different types were continuously isolated. SARS-CoV-2 virus isolated from patient genome analyses results shows SARS-CoV-2 genome, the largest RNA genome in the same betacoronavirus family [18]. SARS-CoV-2 genome consists of 10 genes and two by third is nonstructural gene ORF1ab [19, 20]. The other one by third 3 prime regions of the genome contain four major structural genes such as spike protein, envelope protein, matrix protein, and nucleocapsid protein and five accessory protein ORF3, 6, 7a, 7b, 8, and 10 but ORF10 gene role is not identified [19, 20].

In SARS-CoV-2 virus infection, the spike protein binds to the ACE2 (angiotensin-converting enzyme2) receptor on the surface of the host cell and the TMPRSS2 protein is combined with the spike and ACE2 binding complex [21]. TMPRSS2 cut the intermediate part of the spike protein, virus envelope, and cell membrane fusion start, and then viral genome introduces to cell [22]. ACE2 receptor is expressed on several organs, such as the heart, lungs, kidneys, vascular endothelium, and digestive organs [23].

Just one year ago, SARS-CoV-2 occurred and was transmitted to all over the world and immediately many groups start vaccine research and development. mRNA new type of vaccine rapidly commercialized follow the fast track but absolutely limited time not enough to confirm the efficacy and safety but large scale clinical trial phase 3 like vaccine inoculation start in several countries [24]. Currently, more than 25 different types of COVID-19 vaccines are developed and commercialized. mRNA vaccine already supplied but it has several defects to compare to the conventional vaccine [25]. For example, Vaccine storage condition is -70°C and too short storage period, just two weeks and medical doctor observe vaccine inoculation people more than a half-hour after vaccine injection. Another serious problem is severe pain and fever after vaccination, and these kinds of symptoms continue for more than 3 days and appear in most people. If SARS-CoV-2 transmission occurs continuously, we need more convenient, safe and efficacy vaccines and that vaccine must be easily supplied to Africa, South America, and some middle east Asia including south Asian countries. One another purpose of the vaccine used for disease control is the realization of human equality. So, we must as soon as possible develop cost-effective and more safe and efficacy vaccines to develop and commercialize. Unfortunately, the human race must face fighting New emerging pandemic disease and vaccine is the most important control method, so we also prepare stable and robust more advanced technology for developing vaccine even it was based on previously used technology at this moment. Human must be show overcome as soon as possible finish SARS-CoV-2 pandemic disease.

The viral vector vaccine is one of the good platforms for vaccine development. Viral vector vaccine divides two types, depend on the replicating or non-replicating live virus containing the antigen protein gene or antigen protein. Currently, the adenovirus live viral vector vaccine contains antigen protein gene in the viral genome or plasmid and gene transcription occurred at the nucleus of the host cell, so they have potential risk factors of side effect post-vaccination. Another drawback of the viral vector vaccine is the low-efficiency protein antigen production amount considering the high concentration of inoculated virus. So viral vector vaccines need more improvement in safety issues to make enough protective immunity against virulence virus infection.

Several types of research have been performed on Newcastle disease virus (NDV) using SARS-CoV-2 vaccine development. Most of the study NDV used as an antigen delivery system live or inactivated viral vector. Total spike protein or part of spike protein such as S1 or RBD protein were fused with transmembrane domain and intra-viral domain of the HN or F protein of NDV [26–29]. But they did not mention the mechanism of how to spike protein expressed on the viral surface only using the intergenic sequence of NDV in front of the spike protein gene. One of the research Matrix proteins of NDV used a fusion partner for RBD or spike protein expression. One another research showed spike protein expressed VLP using electron microscopy [30]. Spike protein surface-expressed recombinant NDV virus growth well in Vero 76 cells and showed good immunogenicity. But, they used a not purified antigen in animal tests and did not show surface expression. Spike protein expressed live NDV also made and immunogenicity but contrary to expectation cellular immunity related cytokine expression not increased [31].

In this study, we used a signal peptide of F protein for targeting the host cell membrane because HN and F protein have signal peptides to move the host cell membrane after expression and signal peptide cut off by protease. Another difference to previous researches we used the RBD gene inserted between NP and P gene. In previous studies, Between P and M gene sites is the optimal site for foreign gene expression, but our results show between NP and P gene site are also suitable for foreign gene expression. We established sandwich ELISA for measuring RBD protein amount on the viral surface, and it was also strong proof for RBD protein expressed on the viral surface. Therefore, this may be why high antibody titers were obtained with a small amount of RBD protein on the viral surface.

A new viral vector platform was constructed for protein express on the viral surface and using the RBD domain of the SARS-Cov-2 spike protein. Surface expression platform constructed with NDV genes or some part of the gene of HN or F protein. SARS-CoV-2 RBD gene inserted cDNA of NDV infected to HEp2 mammalian cell, recover recombinant virus, and identified RBD domain express on the surface of recombinant NDV virus. RBD expressed NDV virus tested immunogenicity and safety and show it will be a good candidate vaccine to protect SARS-CoV-2 virus infection. This protein surface expression platform will be used for COVID-19 vaccine production, and this platform is good to rapidly develop new serotype variants. Moreover, this platform will be used for various vaccine development against infectious diseases.

## Materials and methods

### Ethics statement

Animal experiments were conducted in strict accordance with the guidelines of the Ethical Principles and Guidelines for the South Korea of Animals for Scientific Purposes. The animal protocol (Approval number: 2020–03) was approved by the Institutional Animal Care and Use Committee of Libentech co., ltd. Immunization and blood collections were conducted under isoflurane anesthesia. All precautions were made to minimize the suffering to the animals throughout the study.

### Construction of RBD expressing cDNA vector

The viral RNA was extracted by RNA preparation kit (Qiagen, Hilden, Germany) for RNA genome isolation and amplified four fragments by reverse transcription polymerase chain reaction (RT-PCR, Bioneer, Daejeon, South Korea) with the specific 4 pairs primers containing restriction enzyme sites shown in Table 1. RT-PCR was performed by reaction at 42°C for 1 hour and at 94°C for 5 min, followed by a total of 30 cycles of 94°C for 1 min, 60°C for 1 min, and 72°C for 1 min, followed by reaction at 72°C for 7 min. The anti-genomic cDNA construction was sub-cloned to a modified pBR322 low-copy-number plasmid digested Pac I and Pme I restriction enzyme. The pBR322 vector was modified preferably under the control of the T7 RNA polymerase promoter and was positioned so that it was terminated by the hepatitis delta virus (HDV) antigenome ribozyme and T7 terminator gene used to split RNA at the terminus of the NDV genome, thereby allowing viral encapsulation and packaging.

**Table 1:** Primers used in this study for cDNA NDV VG/GA plasmid construction.

Gene	Direction	Sequence (5`→3`)	Restriction site
Fragment 1 (L2)	Forward	ACGCGTggtctcaggtttatatgcagggaa	MluI
	Reverse	TTAATTAAaccaaacaaagatttggtgaatg	PacI
Fragment 2 (L1)	Forward	ACTAGTtgagattctcaaggatgatggggt	SpeI
	Reverse	ACGCGTcgagtgcaagagactaatagtttt	MluI
Fragment 3 (F-HN)	Forward	GGCGCCattatcggtggtgtagctctcgg	Kas I
	Reverse	ACTAGTaaagggacgattctgaattccccg	SpeI
Fragment 4 (P-M-F)	Forward	CCGCGGaaacagccaagagagaccgcagaa	SacII
	Reverse	GGCGCCaaccgggatccagaatcttctacccgt	Kas I
Fragment 5 (NP-P)	Forward	GTTTAAACaccaaacagagaatccgtaagg	PmeI
	Reverse	CCGCGGctttgttgactcccctgttgttga	SacII

**Fig 1:**
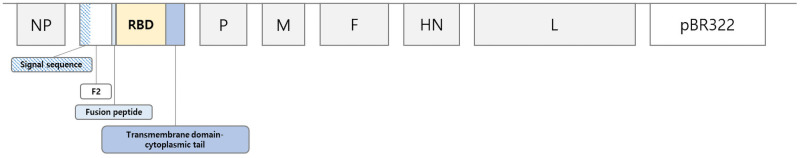
LVP-K1-RBD19 vectors expressing the RBD protein of SARS-CoV-2. Schematic diagram of genome structures of NDV VG/GA expressing RBD protein on the viral surface. The viral surface expression system consisted of the fusion (F) protein. The RBD protein was fused between F2 containing signal sequence and fusion peptide, the transmembrane domain, and the cytoplasmic tail of F1.

The four cDNA fragments have the same nucleotide sequence at the terminus of 15 bp, and a transgene cassette consisting of a GE-IG-GS sequence and multiple cloning site (MCS) was inserted between the NP gene and P gene by overlap cloning to construct the LVP-K1 vector for foreign gene insertion. Four fragments of the NDV cDNA were listed in Table 2. The gene encoding RBD protein was inserted into the NDV surface expression cassette (genes encoding the F2 subunit including signal sequence and HR4, and F1 subunit including fusion peptide, transmembrane domain and cytoplasmic tail of the NDV fusion protein) for surface expression of NDV to construct a SARS-CoV-2 RBD protein expression gene combination (Fig 1). The SARS-CoV-2 RBD protein expression gene combination was designed and synthesized to have a FseI restriction enzyme recognition site and a kozak sequence at the N terminus, and a FseI restriction enzyme recognition site at the C terminus and to be inserted between the NP gene and the P gene of NDV. The LVP-K1 vector for foreign gene insertion and the RBD expression cassette in a ratio of 1: 3, ligation was performed overnight at 4°C using T4 ligase, and the transformation was performed with *E*. *coli* TOP10 competent cells using a heat shock method (heat shock). Then, the seeds were determined through colony PCR using primers, forward primer 5’-GGAACAGGAAGAGAATCAGCAAC-3’ and reverse primer 5’-CAATCTTTCCAGTTTGCCCTG-3’. The plasmid midi preparation was performed to obtain a plasmid expressing the RBD of the SARS-CoV-2 spike protein on the surface of NDV.

**Table 2:** Primers used in this study for the construction of viral vector platform.

Gene	Direction	Sequence (5`→3`)	Size (bp)
Fragment 1 (pBR322-NP)	Forward	TTCTCGCTTCCGGCGGCATC	5,036
	Reverse	CCGCTTCTACCCGTATTTTTTCTAAGCAGAGGAATTGGGATGACCTC	
Fragment 2 (P-M)	Forward	TACGGGTAGAAGCGGCCGCGGCCGGCCCCACACCCCACCCCTCAATCC	2,938
	Reverse	CCGGGATCCAGAATCTTCTACCC	
Fragment 3 (F-HN)	Forward	GATTCTGGATCCCGGTTGGCG	5,578
	Reverse	CCGCCATCACTTGACAGTTCC	
Fragment 4 (L)	Forward	GTCAAGTGATGGCGGAAGGG	5,256
	Reverse	CGCCGGAAGCGAGAAGAATC	

### Recovery of the recombinant virus

NDV transcription complex genes, NP, P, and L were separately cloned to pBR322 vectors and used for helper plasmid (pBR322-NP, pBR322-P, and pBR322-L). Before one day of the transfection, HEp-2 cells (5x10^5^ cells/well) were seeded in 6 well plates. Then, the modified vaccinia virus (MVA-T7) was infected with 1 multiplicity of infection (MOI). 2.5, 1.5, 0.5, and 5 μg of pBR322-NP, pBR322-P, and pBR322-L Helper plasmids expressing proteins by the T7 promoter and LVP-K1-RBD19, a plasmid expressing RBD of SARS-CoV-2 spike protein on the surface were mixed with Lipofectamine 3000 (Invitrogen, Carlsbad, CA, USA) at an appropriate ratio in the cell line to perform their transfection. The HEp-2 cell supernatant was harvested after incubation at 37°C and 5% CO_2_ for 3 to 4 days. Then, the supernatant was inoculated into the allantoic cavity of 9 to 11 days old SPF embryonated egg (Orient Bio, Seongnam, South Korea). The allantoic fluid was collected at 4 days after inoculation. To remove vaccinia virus, allantoic fluid diluted at 10^−3^ with PBS was inoculated into the allantoic cavity of the 9 to 11 days old SPF embryonated egg. After 4 days of inoculation, the allantoic fluid was isolated using a Viral RNA extraction kit (Qiagen). 5 μl of extracted RNA and 1 μl of each of forward and reverse primers in Table 3 were used to react at 42°C for 1 hour, at 94°C for 5 minutes, then perform a total of 35 cycles of at 94°C for 1 minute, at 60°C for 1 minute, and at 72°C for 1 minute, and then react at 72°C for 7 minutes by ONE-STEP RT-PCR (Bioneer).

**Table 3:** Primers used in this study to identify the recovered virus.

Gene	Direction	Sequence (5`→3`)	Size (bp)
NDV check	Forward	CCACAATTCCAAGATAACCGGAG	327
	Reverse	GCTGCCACAATCAGATGCCTTTG	
RBD check	Forward	GTCAGACAAATCGCTCCAGGG	363
	Reverse	AGGTCCACAAACAGTTGCTGG	
Vaccinia virus check	Forward	ATGACGATGAAAATGATGGTACATA	1,059
	Reverse	CTCCAATACTACTGTAGTTGTAAGG	

### Virus concentration and purification

Vero 76 cells were cultured at 3 x 10^5^ cells/ml in 175 cm^2^ t-flask. On the next day, the recombinant virus was inoculated at 0.05 MOI for 2 days following general virus inoculation method [32]. The virus titer was measured at the 2 days post-inoculation. The viruses were clarified by centrifugation at 5,000 g, at 4°C for 10 min to remove debris and the supernatant was collected. The virus was pelleted by ultracentrifugation (rotor No 9, Ultra 5.0, Hanil, Daejeon, South Korea) at 32,000 rpm for 3 hours at 4°C. The supernatants were aspirated off, and the virus pellet was re-suspend in TNE Buffer (10 mM Tris-HCl, 20 mM NaCl, 1 mM EDTA). The concentrated virus was purified at ultracentrifuge in the 10 to 40% sucrose gradient for 3 hours at 32,000 rpm 4°C. The purified virus was received in 1 ml fractions and measured UV absorption at 260 and 280 nm [33]. The fraction was used to virus titration following TCID50 measurement titration method. The purified virus fraction was dialyzed using dialysis tubing cellulose membrane (33 mm, lot 3110, Sigma Aldrich, St. Louis, MO, USA) against PBS buffer (pH 7.4) at 4°C overnight.

### Identification of the RBD viral surface expression

BCA protein analysis was used to measure the protein concentration. Then, 50 μg of protein was separated through 10% SDS-PAGE and transferred on a PVDF membrane. The membranes were reacted with the SARS-CoV-2 Spike RBD Antibody (R&D Systems, Minneapolis, MN USA) and NDV HN protein Polyclonal Antibody (Bioss Antibodies, Woburn, MA, USA) in blocking buffer at room temperature for 1 hour following general western blotting method, followed by goat anti-rabbit IgG-HRP (Invitrogen). Reactive proteins were detected with ECL kit (Invitrogen) following the manufacturer’s protocol.

### Virus inactivation

LVP-K1-RBD19 virus sample was taken and the virus titer was measured according to the general TCID50 measurement method, and the titer was adjusted using PBS according to the virus titer. After diluting the virus titer to 10^8.0^ TCID_50_/ml, 0.1% aqueous formalin was added thereto. They were stored at 4°C for 48 hours. After inactivation, samples were collected at 6-, 12-, 24-, 36-, and 48-hours using Vero 76 cells. To observe CPE caused by virus infection, the titer was observed using the TCID50 method for 5 days to confirm inactivation.

### Establishment of the sandwich ELISA

The sandwich ELISA method was used to determine the concentration of the surface-expressed RBD protein. SARS-CoV-2 spike protein polyclonal antibody (Invitrogen) was diluted to a concentration of 1:1000 in carbonate-bicarbonate buffer (40 mmol/L Na_2_CO_3_, 60 mmol/L NaHCO_3_, pH 9.6). Then, a 96-well plate was coated with the coating buffer at 4°C. In next day, the coating buffer was discarded, and the plate was blocked using 1% BSA diluted in PBST (0.1% tween20, pH 7.4). The inactivated virus (10^7.7^ TCID_50_/ml) and RBD protein (H-GUARD, Deajeon, South Korea) as a positive control were serially diluted 10-fold using PBS. 100 μl/well of the virus sample or RBD protein was added and incubated at 37°C for 1 hour. Anti-SARS-CoV-2 spike RBD antibody (R&D Systems, 1 μg/ml) diluted in PBS at 1:1000 after washing five times with PBST (PBS containing 0.5% Tween20) was treated at 100 μl/well at 37°C for 1 hour. After washing with PBST five times, an anti-mouse IgG secondary antibody labeled with horseradish peroxidase (HRP) was treated at 1:5000 at 37°C for 1 hour according to the manufacturer’s instructions. The TMB substrate solution was treated at 100 μl/well and then cultured at room temperature for 30 minutes in the dark. The reaction was stopped by adding 50 μl/well of 2M H_2_SO_4_. OD was measured at 450 nm using a microplate reader (iMark, Bio-Rad, Hercules, CA, USA).

### Immunogenicity test of the inactivated virus

The 7-week-old, female BALB/c mice were obtained from the Orient Bio and vaccinated with 1 μg/100 μl, 5 μg/100 μl, and 10 μg/100 μl of RBD protein expressed on the inactivated LVP-K1-RBD19 virus, the recombinant RBD protein (10 μg/100 μl) with alum, and PBS (100 μl) through the intramuscular route. A total of 20 mice have randomly divided into five groups and each group has four mice. One group inoculated RBD proteins 10 μg with Alum was used as a positive control. The group inoculated PBS was used as a negative control group. The intramuscular inoculation was performed twice at an interval of 2 weeks at an amount of 100 μl/dose. Blood was collected before the first vaccination, at 14 and 28 days post-inoculation. The blood samples were collected from the retro-orbital sinus after anesthesia. The serum was separated and inactivated at 56°C for 30 min. ELISA test following general indirect ELISA test protocol. 100 ul (2 μg/ml) of the RBD protein (H-GUARD) in tris buffer (20 mM, pH 8.0) was plated in the 96-well plates and incubated at 4°C overnight. After discarding the solution, the 96-well plate was added 200 μl of blocking buffer (1.0% BSA containing Tris Buffer pH 8.0) and incubated at room temperature for 1 hour. After removing the solution, the plate was washed three times with 200 μl tris buffer containing 0.5% tween 20. 1 in 2 diluted serum samples was diluted two-fold with the same volume of tris buffer, added to 96-well plates, and incubated at room temperature for 1 hour. After removing the serum samples, the plate was washed three times with washing buffer. 100 μl of diluted horseradish peroxidase (HRP)-conjugated goat anti-mouse IgG antibodies were added to the wells and incubated at 37°C for 1 hour. After three wash steps with washing buffer, 50 μl of TMB substrate solution (Thermo Scientific), and samples were incubated at 37°C for 15 min. The reaction was terminated by adding 50 μl of 1 M sulfuric acid, and OD was measured at 450 nm using a microplate reader (iMark, Bio-Rad).

### Pseudovirus neutralization assay

The pseudovirus of SARS-CoV-2 was constructed using an HIV-based system (HIV-2019-nCoV-spike pps-myc-Luc). The virus-neutralizing (VN) antibody titer test was performed using a pseudovirus and tested in the group of 10 μg of RBD protein expressed on the surface of LVP-K1-RBD19 virus and recombinant RBD protein with alum as a positive control. The serum sample was diluted in 96 well plates by a two-fold dilution method with PBS. The equal volume (100 μl) of pseudovirus (virus titer: 1x10^3.0^ TCID_50_/ml) was mixed and reacted for 1 hour at room temperature. The reaction was finished and the pseudovirus mixture was transferred to monolayer-formed Huh7 cells in the 96 well plate and incubated for 1 hour at room temperature. After finishing incubation, mixture solution discarded and one-time washed with media and added 100 μl of media to incubate for 18 hours at 37°C, 5% CO_2_ incubators. The fluorescence (Fluc) emitted from cells was measured using the bright Glo luciferase assay system (Promega, Madison, WI, USA). After discarding the media, 50 μl of luciferase lysis buffer was added to the well and store at -70°C for 24 hours. Then, luciferase reagent was added and the Relative light unit (RLU) was measured. RLU measure was using GLOMAX illuminometer. The median neutralization dose was calculated by the Reed-Munch method.

### Statistical analysis

All data showed as the mean ± the standard error of the mean (SEM), as indicated. Statistical comparisons were identified by using Student’s t-test or one-way ANOVA with SPSS (Version 23, SPSS, Inc., Chicago, IL, USA). P-values < 0.05 indicated statistically significant.

## Results

### Generation of NDV VG/GA expressing the SARS-CoV-2 RBD protein

The complementary DNA of the NDV VG/GA strain was amplified to four cDNA fragments by the reverse-transcribed PCR (Fig 1A and Table 1). Helper plasmids, pBR322-NP, pBR322-P, and, pBR322-L, were constructed and verified by DNA sequencing. To identify the accurate role of helper plasmids were co-transfected with the cDNA of NDV VG/GA strain into HEp-2 cells [14]. In addition, the multiple cloning site (MCS) was inserted between NP and P gene (LVP-K1) using an overlap cloning system for the expression of the additional gene. In this study, the RBD protein is the major antigen of SARS-CoV-2 for protective immunity. To express RBD protein by the NDV VG/VA strain, a transcriptional unit encoding viral surface expression cassette was inserted at the NP and P junction of the VG/GA antigenomic cDNA.

**Fig 2:**
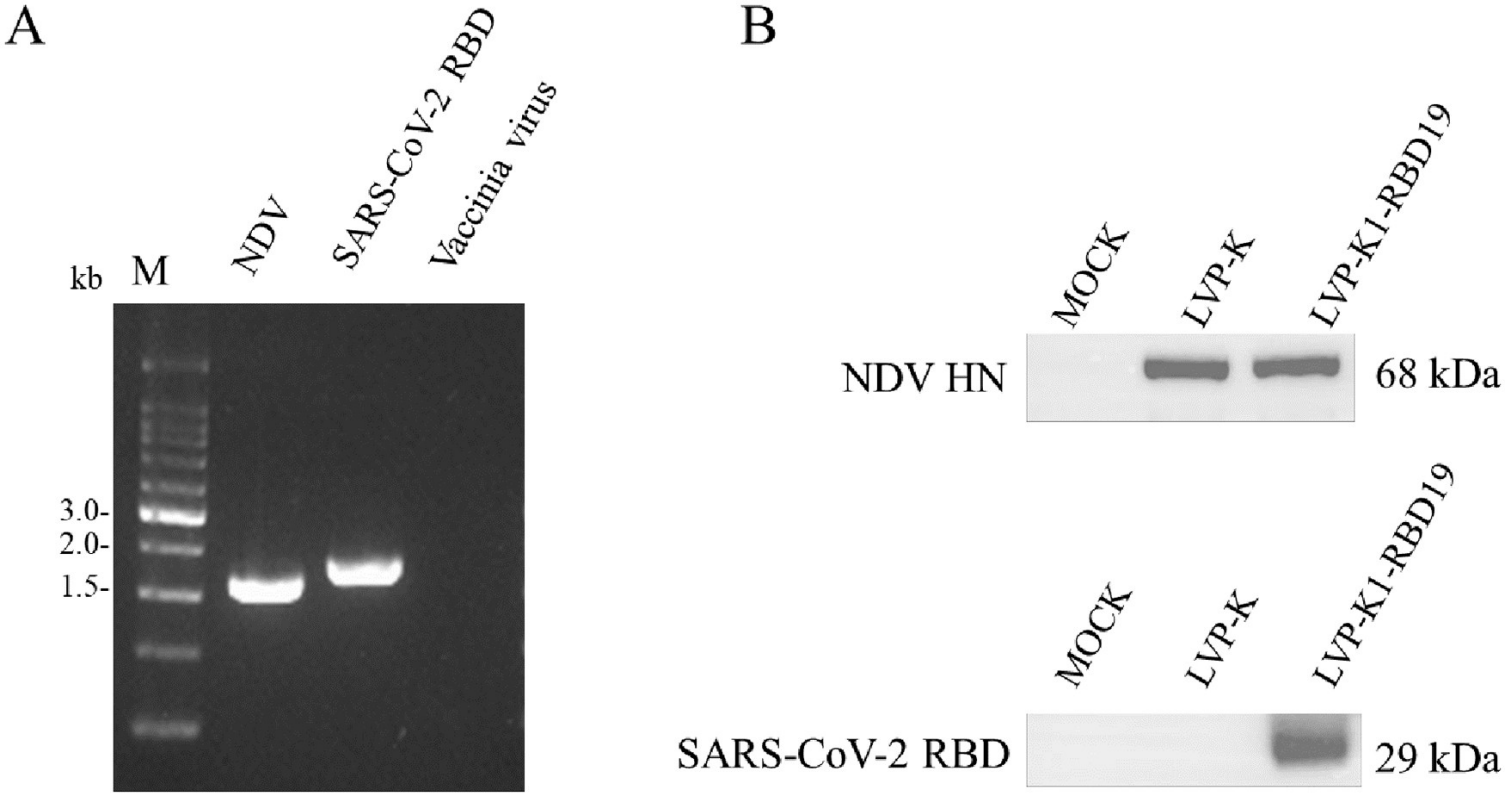
Identification of LVP-K1-RBD19 virus. (A) Inserted RBD gene with transgene expression cassette identified by RT-PCR. (B) LVP-K1-RBD19 recombinant virus expressed RBD protein on the viral surface and LVP-K1 virus did not show RBD protein.

LVP-K1-RBD19 virus was rescued by transfection of HEp-2 cells. The virus amplified in embryonated chicken eggs. The harvested virus was diluted ten folds dilution (10^−1^~10^−5^) and inoculated in embryonated chicken eggs for removing vaccinia virus. The virus removed vaccinia virus was inoculated in eggs for the virus amplification. In Fig 2A, vaccinia virus was removed in the SPF egg. RT-PCR and sequence analysis of the rescued virus was identified as we designed (Table 3). All the viruses expressing the RBD protein grew to high titers (~10^7^ TCID_50_/ml) in Vero 76 cells, which is beneficial for the production of a low-cost vaccine. These results determined that the LVP-K1-RBD19 virus was recovered successfully.

### Identification of RBD protein on the virus surface

To evaluate the expression of RBD protein, Vero 76 cells were infected with LVP-K1-RBD19 virus at an MOI of 0.1 for 2 days. LVP-K1 virus was also cultured the same as LVP-K1-RBD19 virus culture. The LVP-K virus was used as the negative control. 20 μg of the Purified virus sample processed by SDS-PAGE and separated protein transfer to western blotting membrane. To examine the RBD protein on the surface of NDV, the virus was analyzed as an immunoblot assay using SARS Coronavirus Spike Protein Polyclonal Antibody (Invitrogen). The expression of the HN protein was detected as a control of the virus (Fig 2C). This result show LVP-K1-RBD19 expressed RBD protein and any problem in RBD expressed recombinant virus propagation in Vero 76 cell culture. The results demonstrated that the F2 containing signal sequence, cleavage site, and, TM/CT in F1 of NDV F protein in LVP-K1-RBD19 facilitates the membrane-anchoring of the RBD as expected.

### Establishment of the sandwich ELISA

**Fig 3:**
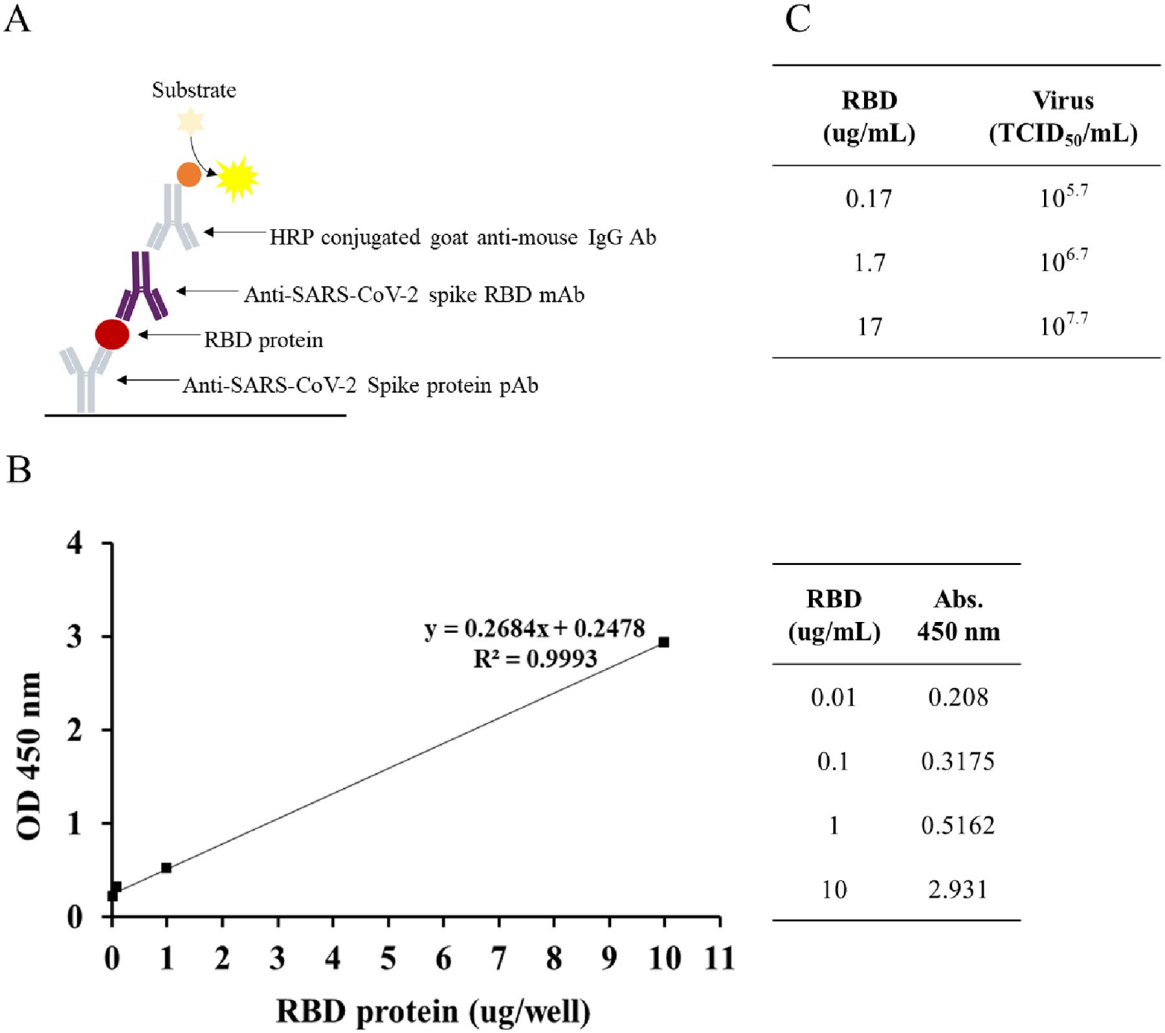
Establishment and validation of a sandwich ELISA for RBD protein quantification on LVP-K1-RBD19 virus. (A) Schematic diagram of the developed sandwich ELISA. (B) Standard curve of the developed sandwich ELISA for quantification of RBD protein. The data was identified to assess the correlationship between RBD protein and the RBD protein of LVP-K1-RBD19 (r^2^ = 0.9993). (C) Optical density (OD) of the 10^5.7^ TCID_50_/100 μl of LVP-K1-RBD19 virus is 0.294. This OD put into equation of the standard curve, the amount of RBD protein is a 0.17 μg.

Viral surface-expressed RBD protein concentrations measured by sandwich ELISA method (Fig 3A). Spike protein-specific Polyclonal antibody capture RBD expressed virus and RBD protein specific monoclonal antibody bind virus through RBD protein on the viral surface. Optical density (OD) at 450 nm depends on the amount of horseradish peroxidase-conjugated antibody correlated with RBD binding monoclonal antibody therefore RBD concentration of the viral surface-expressed calculated based on the recombinant RBD protein used as a standard graph. Fig 3B shows standard curve OD depends on the RBD protein concentration. Standard curve of the developed sandwich ELISA for quantification of RBD protein. The data were identified to assess the correlations between RBD protein and the RBD protein of LVP-K1-RBD19 (r^2^ = 0.9993). The OD of the 10^5.7^ TCID_50_/100 μl of LVP-K1-RBD19 virus is 0.294. This OD value was put into the equation of the standard curve, the amount of RBD protein was 0.17 μg (Fig 3C). The RBD protein amount, expressed on the surface of the inactivated LVP-K1-RBD19, was measured 0.17 μg per 10^5.7^ TCID_50_/ml, and total viral protein amount of LVP-K1-RBD was measured 9.4 μg per same virus concentration. Normally LVP-K1-RBD19 virus titer reaches 10^8.1^ TCID_50_/ml using Vero 76 cell line, we produce more than 20 μg/ml of RBD on the viral surface. Sandwich ELISA result was another strong proof of the RBD expression on the viral surface because sandwich ELISA was done by a specific antibody reaction between RBD and Antibody.

### Immunogenicity and safety test of the inactivated LVP-K1-RBD19 virus

**Fig 4:**
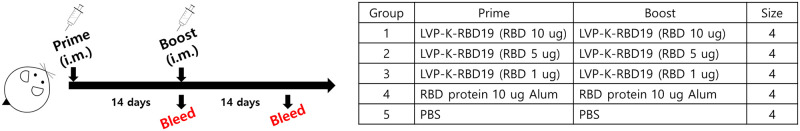
Immunization schedule and groups. BALB/c mice were intramuscularly vaccinated at a 2-week interval. Mice were bled at 14 and 28 days post-inoculation (dpi) for in vitro serological assays. Group 1, 2, and 3 were inoculated with 1, 5, and 10 μg of RBD protein expressed on the surface of LVP-K1-RBD19, respectively; group 4 was inoculated with 10 μg of the RBD protein combined with Aluㄹm; group 5 was inoculated with PBS as a negative control.

**Fig 5:**
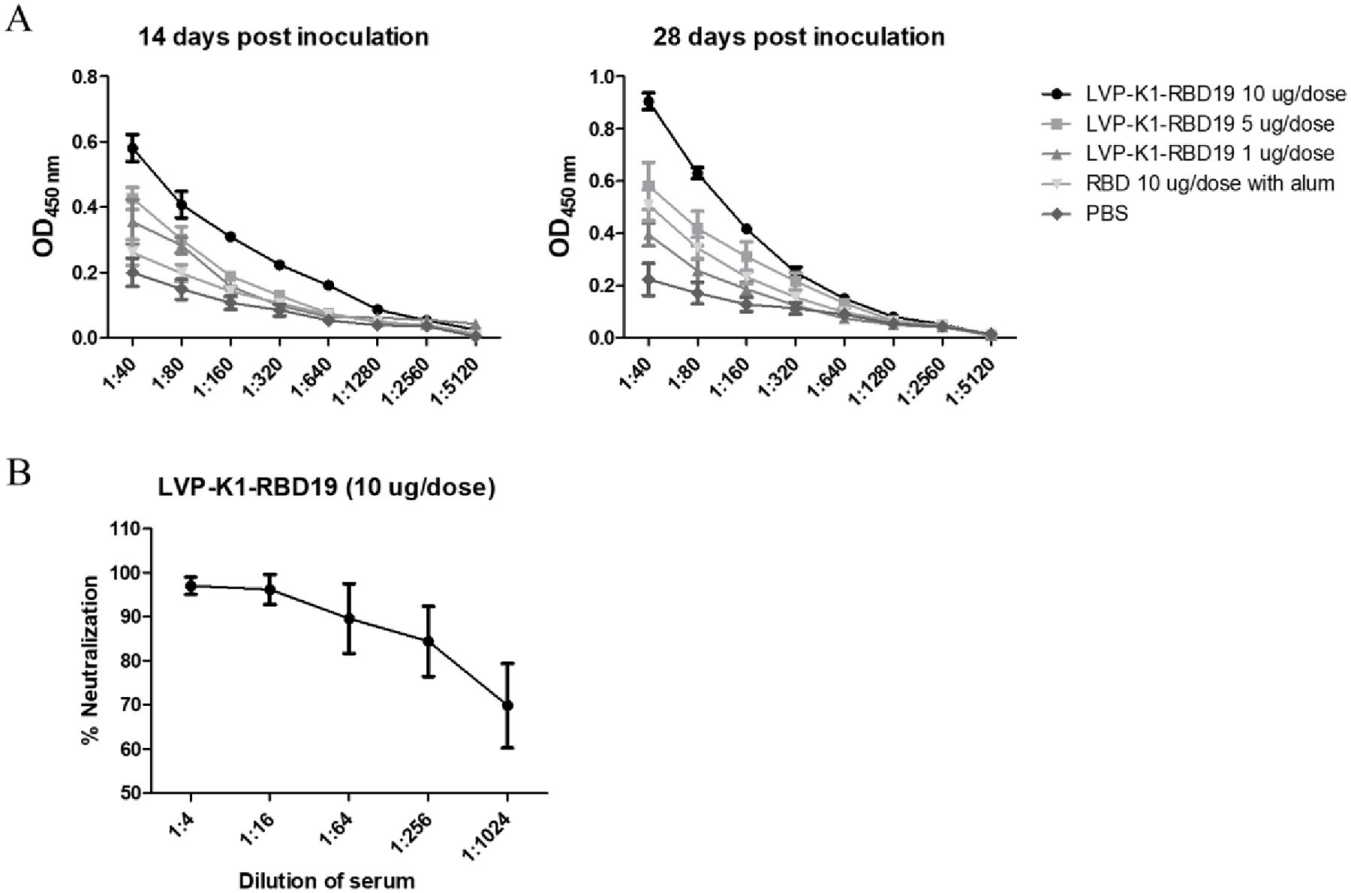
Inactivated LVP-K1-RBD19 virus induces high antibody responses in mice. (A) RBD-specific serum IgG titer by an enzyme linked immunosorbent assay (ELISA). Serum IgG titers from mice after 14 and 28 dpi were determined to the RBD specific antibody by 2-fold serial dilution from 1:8 to 5,120. (B) Neutralization titers of serum antibodies. Pseudovirus neutralization assays were conducted to determine the neutralizing antibodies from mice vaccinated 10 μg of RBD protein expressed on the surface of LVP-K1-RBD19 after 28 dpi.

Inactivated LVP-K1-RBD19 virus used for making laboratory trial vaccine for testing immunogenicity and safety using mouse (Fig 4). Mice were bled pre-primary inoculation, 14 (boost inoculation) and 28 days after primary vaccination to detect RBD specific antibody responses by ELISAs (Fig 5A), and 28 days pseudovirus-neutralization assays (Fig 5B). Alum adjuvant mixed RBD protein 10 μg as a positive control. Mice-inoculated PBS was used as a negative control. Fig 5A showed 5 μg/dose inactivated LVP-K1-RBD19 total IgG titer show almost same result with alum mixed 10 μg of RBD for the unadjuvanted at 14 dpi. Especially 10 μg/dose RBD protein expressed on the surface of inactivated LVP-K1-RBD19 virus inoculating group show total IgG antibody titer reaches to saturation level two-week post first dose inoculation. The antibody titers of the LVP-K1-RBD19 groups inoculated at 1 and 5 μg increased after boost inoculation. On the other hand, immunization with 10 μg of RBD protein expressed on the surface of LVP-K1-RBD19 induced the high antibody titer at 14 dpi. The boost significantly increased the antibody titers of all LVP-K1-RBD19 immunization groups and RBD 10 μg with the alum group at 28 dpi. Inoculation with 10 μg of RBD protein formulated with Alum and 1 and 5 μg of RBD protein expressed on the surface of inactivated LVP-K1-RBD19 virus without adjuvant-induced similar levels of the RBD specific antibody at 28 dpi, which is comparable to the titers induced by 10 μg of RBD protein expressed on the surface of LVP-K1-RBD19 virus at 14 dpi. The neutralization assays were performed to identify the neutralizing antibodies of serum from 10 μg of RBD protein expressed on the surface of LVP-K1-RBD19 virus inoculated mice. Sera from all mice inoculated with 10 μg of RBD protein expressed on the surface of LVP-K1-RBD19 detected neutralizing activity against the SARS-CoV-2 pseudotyped virus. Thus, 10 μg of RBD protein expressed on the surface of LVP-K1-RBD19 can be effective at eliciting neutralizing titers at 28 dpi compared with the RBD 10 μg with the alum group (S1 and S2 Figs). These data significantly demonstrated that inactivated LVP-K1-RBD19 virus can induce neutralizing activities against SARS-CoV-2 in a short period and could be an excellent candidate for COVID-19 vaccine development.

## Discussion

The outbreak of COVID-19 pandemic disease impact worldwide serious damage occurred human health and the economy. Moreover, in the 21st century, medical science and biotechnology have been advanced significantly compare to the past 14st Pest and 20st Spanish influenza, the new emerging disease still very dangerous to human nature. Several vaccines rapidly developed and vaccines have been provided to limited people but vaccines have several side effect even fatal side effects case reported [34] and the exact ability of the vaccine to protect against viral infection remain as a subject of investigation. Moreover, unstable vaccine antigen needs very sophisticated logistics and short self-life need several complex considerations for proper distribution. Another big challenge to overcome SARS-CoV-2 pandemic needs rapid vaccine development technology following new genetic variant appearance [35]. Therefore, the new preventive vaccine against SARS-CoV-2 should have several improvements as well as efficacy. More stable antigen, more effective to prevent, fewer side effects, and rapid new antigen application. In these considerations. Antigen surface-expressed non-replicating viral vector vaccine will be able to a new alternative vaccine type against SARS-CoV-2.

This study shows a new type of vaccine, combined inactivated vaccine and viral vector vaccine have several improvements compared to the currently used vaccine. Viral surface expression of the RBD protein of SARS-CoV-2 on the avirulent NDV virus could be a good candidate vaccine to prevent rapid change and the fast-transmitted new genetic variant of SARS-CoV-2. RBD expression NDV viral vector has antigen expression cassette between NP and P gene, which used for foreign gene expression on the viral surface of NDV. The Antigen expression cassette consists of several genes of the Fusion protein of the NDV. Fusion protein expression related to several genes also working foreign protein gene expression without any kind of influence on virus growth and foreign protein expression. It is the first successful result of RBD protein expression through surface expression cassette consist of IGS sequence of P gene and several genes of F protein of NDV. Previously most of the foreign gene expression using the site between P and M gene, which site is the optimal site for foreign gene expression site. This study also using the NP and P gene site (LVP-K1-RBD19 (NP/P)) and compare with RBD expression amount and recombinant virus growth P and M gene site (LVP-K1-RBD19 (P/M)), but there is not a big difference. The results were shown in S3–S5 Figs. Therefore, several studies must use a new foreign gene insertion site in the NDV for finding the best site. We must consider RNA transcription level depend on the distance from the 3’ end of NDV RNA genome. IGS sequence is also deeply related to structural gene expression and foreign gene expression of each structural gene of NDV. M protein concentration is the critical changing point transcription and translation of NDV to make progeny virus particles. So now we must do find out optimal conditions considering the distance from 3’, IGS sequence, and M protein concentration as an important factor for foreign gene expression in NDV.

The F protein gene encodes a protein of 553 amino acids. F protein is compounded as an inactive precursor, F0, that is activated by proteolytic cleavage to the competent form (the disulfide-linked F1-F2 fusion) [36–42], F2 including the signal sequence and cleavage site and F1 including fusion peptide, transmembrane domain (TM), and cytoplasmic domain (CT). A viral surface expression cassette was developed that included a cleavage site as well as a signal sequence, TM and CT. Since the signal sequence of F protein cleaved at the cell membrane for flip over anchoring outside of cell membrane. F protein signal peptide co-expressed with RBD protein also cleave occurred at the cell membrane and RBD protein anchoring at the cell membrane using Transmembrane and intra-viral tail sequence and then new synthesized NDV particle assembled and cellular membrane take out as a viral envelope during the budding. RBD protein is expressed on the viral surface with the hydrophobic interaction between the F protein transmembrane domain and the viral envelope. It is the first study of several genes of the F protein using surface expression cassette design and used for foreign antigen protein expression on the NDV surface produced as a non-replicating viral vector vaccine.

RBD protein on the surface of NDV was named LVP-K1-RBD19. LVP-K1-RBD19 successfully showed no change in growth kinetics in Vero 76 cells which is means that the RBD protein is expressed on the viral surface in no conflict with HN and F protein expression. The mice receiving inactivated LVP-K1-RBD19 virus twice intramuscularly have enhanced high levels of RBD-specific antibodies. The same concentration of the surface-expressed RBD protein show more high total IgG titer compares with the same concentration of the recombinant RBD mixed with aluminum hydroxide gel. Serum sample using virus neutralization antibody titration result show 80% neutralization at the VN titer is 27. NDV as a viral vector, have several biomolecules stimulating TLRs (Toll-like receptor) and TLR related immune response enhancing humoral immunity as well as cellular immunity [43]. RBD protein exposes to the immune system and stimulates humoral immune response electing several types of IgG. At the same time, NDV viral vectors were also exposed to the immune system depend on their molecules. For example, single-stranded NDV genomic RNA recognized by TLR7 expressed on the dendritic cell and TLR7 activate DC start signal transduction of immune response especially Type I interferon or MyD88-NF-κB dependent inflammatory cytokine [44]. Interleukin 1, interleukin 12, and tumor necrosis alpha-like inflammatory cytokine activate monocyte and macrophage. Another immunological advantage of using NDV as a viral vector is that inactivated NDV working like a VLP in which has been proved to be useful as it could elicit both humoral and cell-mediated immune responses in immunized hosts. Moreover, VLPs mimics the structure of the wild-type virus and could be recognized by the host immune system as well as RBD protein. For instance, RBD expressed inactivated NDV stimulated the maturation of dendritic cells, upregulated the expression of MHCII, CD40, CD80, and CD86 and cytokine secretions including- TNF-α, IFN-γ, IL-6, IL-4, and IL12p70 [45]. The induction of IgG response and the presence of CD4^+^, CD8^+^ T cells indicate the efficiency of RBD surface-expressed NDVs in inducing humoral and cellular immune responses.

Spike protein have more epitope than RBD domain but Whole spike protein need modification of cleavage site existing between S1 and S2 subunit. Whole spike protein molecular weight reach to 180 kDa it means too big protein expressed on the viral surface so in this study only RBD protein expressed on the viral surface. More important considerations, most of the genetic variants that can affect vaccine efficacy, such as Delta and Delta plus, critical mutations occurred in the RBD gene and it is more advantageous to use RBD for the fast development of vaccines to counter such mutations occurred genetic variant. A study of RBD and spike protein used a vaccine development, report by Yang J. et al. [46]. demonstrated that an RBD-based COVID-19 vaccine can induce much higher titers of neutralizing antibodies than the S protein and S1 subunit. In this study show RBD-based vaccines can effectively protect hACE2-knock-in mice against SARS-CoV-2 challenge. But in SARS-CoV-2 need more study about epitopes related with humoral and cellular immune response. In other word, more precisely, how much T cell epitopes will affect to vaccine efficacy is need more sophisticated Immunological Studies study.

This study showed that dose-dependent antibody responses, which were observed for different doses (1, 5 and 10 μg). We do not check cellular immune response but it will be studied in the future. Nevertheless, this paper importantly supports that the RBD surface-expressed inactivated NDV vaccines were shown better than recombinant RBD subunit vaccine and this result encouraging NDV vector using vaccine technology use develop against new emerging diseases. RBD protein surface-expressed on NDV viral vector vaccine is an original first technology, rapid vaccine development, and alternative to other SARS-CoV-2 vaccines that can be manufactured using the existing facility in cost-effective vaccine production and vaccine campaign include developing countries. This study using viral vector vaccine used RBD domain of 2019 isolated SARS-CoV -2 virus, so it must be tested cross protection against delta or omicron-like genetic variants. We need to prepare a more efficacious vaccine against a new emerging genetic variant strain because we will confront another genetic variant SARS-CoV-2.

## Supporting information

S1 Fig Pseudovirus neutralization titers of serum antibodies. Pseudovirus neutralization assays were conducted to determine the neutralizing antibodies from mice vaccinated 10 μg of RBD protein expressed on the surface of LVP-K1-RBD19 and 10 μg of RBD protein with alum after 28 days post-inoculation.
(DOCX)

S2 Fig Neutralization titers of serum antibodies. hACE2 transgenic mouse 5 groups were randomly divided into five mice in 5, 10 μg of RBD protein expressed on the surface of LVP-K1-RBD19, and Negative control and one mouse in the positive control (RBD and Spike). The positive control group was inoculated with RBD or spike protein three times. The 10 μg of RBD and spike protein were inoculated with the proteins mixed with complete at first immunization, incomplete adjuvant at second in two weeks intervals through intramuscular injection, and 10 μg of RBD without adjuvant at one week post second immunization through tail vein injection. LVP-K1-RBD19 5 and 10 μg were inoculated twice without adjuvant with an interval of 14 days.
(DOCX)

S3 Fig Growth kinetics of LVP-K1-RBD19 (NP/P), LVP-K1-RBD19 (P/M), and LVP-K1 viruses in Vero 76 cells. Cells were infected at an MOI of 0.1, and supernatants were collected at the indicated time points. Viral titers were determined by TCID50 titration on Vero 76 cells.
(DOCX)

S4 Fig RBD protein expression level identified by Western blotting. Vero 76 cells were harvested 24 h post-infection with LVP-K1 infected control cells (lane 1), LVP-K1-RBD19 (NP/P), and LVP-K1-RBD19 (P/M) infected cells. Supernatant was subjected to SDS-PAGE, blotted onto PVDF membranes, and incubated with anti-SARS-CoV-2 RBD mouse monoclonal antibodies. The RBD protein expression levels of the LVP-K1-RBD19 (NP/P) virus were higher than that in the LVP-K1-RBD19 (P/M) virus.
(DOCX)

S5 Fig RT-PCR analysis of LVP-K1-RBD19 virus. The NDV gene was amplified by RT-PCR from total RNA of the infected cells of the indicated virus passages (1 to 10) using the oligonucleotides in Table 3. The band size of a DNA ladder is indicated on the left side.
(DOCX)

S6 Fig Sequence alignment of RBD gene. The genome was analyzed to (A) LVP-K1-RBD19 (NP/P) virus in passages 1 and 7 and (B) LVP-K1-RBD19 (P/M) in passages 1 and 7.
(DOCX)

S1 Text Neutralization assay. (DOCX)

S2 Text Comparison of LVP-K1-RBD19 (NP/P) & LVP-K1-RBD19 (P/M) viruses. (DOCX)

S3 Text (DOCX)

S1 Raw images (TIF)

S2 Raw images (TIF)
