# Supplementary material for: The human ACE-2 receptor binding domain of SARS-CoV-2 express on the viral surface of the Newcastle disease virus as a non-replicating viral vector vaccine candidate
Source: PLoS One. 2022 Feb 8;17(2):e0263684. doi: 10.1371/journal.pone.0263684 (PMC8824364; doi:10.1371/journal.pone.0263684)
Supplement: S1 Fig — Pseudovirus neutralization assays were conducted to determine the neutralizing antibodies from mice vaccinated 10 μg of RBD protein expressed on the surface of LVP-K1-RBD19 and 10 μg of RBD protein with alum after 28 days post-inoculation. (DOCX) [file pone.0263684.s001.docx]

S1 Fig.





**S1 Fig. Pseudovirus neutralization titers of serum antibodies.** Pseudovirus neutralization assays were conducted to determine the neutralizing antibodies from mice vaccinated 10 μg of LVP-K1-RBD19 and 10 μg of RBD protein with alum after 28 days post-inoculation.
