# Supplementary material for: The human ACE-2 receptor binding domain of SARS-CoV-2 express on the viral surface of the Newcastle disease virus as a non-replicating viral vector vaccine candidate
Source: PLoS One. 2022 Feb 8;17(2):e0263684. doi: 10.1371/journal.pone.0263684 (PMC8824364; doi:10.1371/journal.pone.0263684)
Supplement: S2 Fig — hACE2 transgenic mouse 5 groups were randomly divided into five mice in 5, 10 μg of RBD protein expressed on the surface of LVP-K1-RBD19, and Negative control and one mouse in the positive control (RBD and Spike). The positive control group was inoculated with RBD or spike protein three times. The 10 μg of RBD and spike protein were inoculated with the proteins mixed with complete at first immunization, incomplete adjuvant at second in two weeks intervals through intramuscular injection, and 10 μg of RBD without adjuvant at one week post second immunization through tail vein injection. LVP-K1-RBD19 5 and 10 μg were inoculated twice without adjuvant with an interval of 14 days. (DOCX) [file pone.0263684.s002.docx]

S2 Fig.


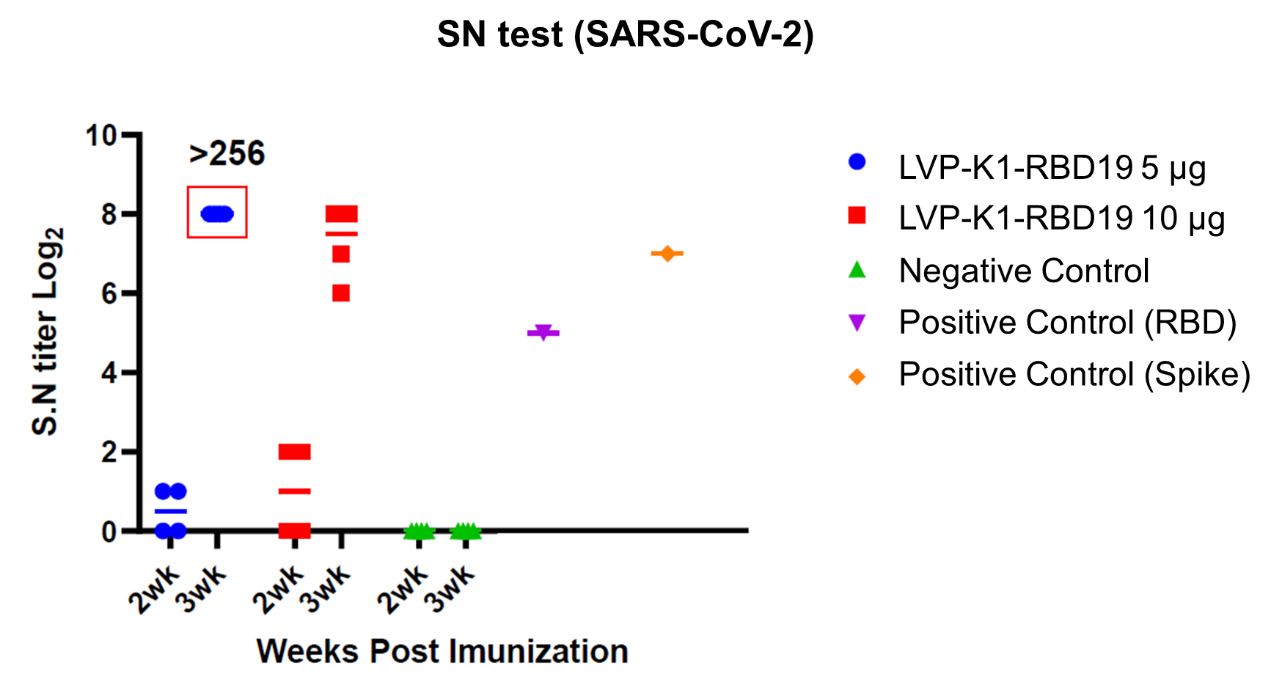


**S2 Fig. Neutralization titers of serum antibodies.** hACE2 transgenic mouse 5 groups were randomly divided into five mice in LVP-K1-RBD19 5, 10 μg, and Negative control and one mouse in the positive control (RBD and Spike). The positive control group was inoculated with RBD or spike protein three times. In the case of the first inoculation, the 10 μg of RBD and spike protein were mixed with complete at first immunization and incomplete adjuvant at second and third immunization in 2-week intervals. LVP-K1-RBD19 5 and 10 μg were inoculated twice without adjuvant with an interval of 14 days.
