# Supplementary material for: The human ACE-2 receptor binding domain of SARS-CoV-2 express on the viral surface of the Newcastle disease virus as a non-replicating viral vector vaccine candidate
Source: PLoS One. 2022 Feb 8;17(2):e0263684. doi: 10.1371/journal.pone.0263684 (PMC8824364; doi:10.1371/journal.pone.0263684)
Supplement: S3 Fig — Cells were infected at an MOI of 0.1, and supernatants were collected at the indicated time points. Viral titers were determined by TCID50 titration on Vero 76 cells. (DOCX) [file pone.0263684.s003.docx]

S3 Fig.





**S3 Fig. Growth kinetics of LVP-K1-RBD19 (NP/P), LVP-K1-RBD19 (P/M), and LVP-K1 viruses in Vero 76 cells.** Cells were infected at an MOI of 0.1, and supernatants were collected at the indicated time points. Viral titers were determined by TCID50 titration on Vero 76 cells.
