# Supplementary material for: The human ACE-2 receptor binding domain of SARS-CoV-2 express on the viral surface of the Newcastle disease virus as a non-replicating viral vector vaccine candidate
Source: PLoS One. 2022 Feb 8;17(2):e0263684. doi: 10.1371/journal.pone.0263684 (PMC8824364; doi:10.1371/journal.pone.0263684)
Supplement: S4 Fig — Vero 76 cells were harvested 24 h post-infection with LVP-K1 infected control cells (lane 1), LVP-K1-RBD19 (NP/P), and LVP-K1-RBD19 (P/M) infected cells. Supernatant was subjected to SDS-PAGE, blotted onto PVDF membranes, and incubated with anti-SARS-CoV-2 RBD mouse monoclonal antibodies. The RBD protein expression levels of the LVP-K1-RBD19 (NP/P) virus were higher than that in the LVP-K1-RBD19 (P/M) virus. (DOCX) [file pone.0263684.s004.docx]

S4 Fig.





**S4 Fig. RBD protein expression level identified by Western blotting.** Vero 76 cells were harvested 24 h post-infection with LVP-K1 infected control cells (lane 1), LVP-K1-RBD19 (NP/P), and LVP-K1-RBD19 (P/M) infected cells. Supernatant was subjected to SDS-PAGE, blotted onto PVDF membranes, and incubated with anti-SARS-CoV-2 RBD mouse monoclonal antibodies. The RBD protein expression levels of the LVP-K1-RBD19 (NP/P) virus were higher than that in the LVP-K1-RBD19 (P/M) virus.
