# Supplementary material for: The human ACE-2 receptor binding domain of SARS-CoV-2 express on the viral surface of the Newcastle disease virus as a non-replicating viral vector vaccine candidate
Source: PLoS One. 2022 Feb 8;17(2):e0263684. doi: 10.1371/journal.pone.0263684 (PMC8824364; doi:10.1371/journal.pone.0263684)
Supplement: S5 Fig — The NDV gene was amplified by RT-PCR from total RNA of the infected cells of the indicated virus passages (1 to 10) using the oligonucleotides in Table 3. The band size of a DNA ladder is indicated on the left side. (DOCX) [file pone.0263684.s005.docx]

S5 Fig.





**S5 Fig. RT-PCR analysis of LVP-K1-RBD19 virus.** The NDV gene was amplified by RT-PCR from total RNA of the infected cells of the indicated virus passages (1 to 10) using the oligonucleotides in table 3. The band size of a DNA ladder is indicated on the left side.
