# Supplementary material for: The human ACE-2 receptor binding domain of SARS-CoV-2 express on the viral surface of the Newcastle disease virus as a non-replicating viral vector vaccine candidate
Source: PLoS One. 2022 Feb 8;17(2):e0263684. doi: 10.1371/journal.pone.0263684 (PMC8824364; doi:10.1371/journal.pone.0263684)
Supplement: S6 Fig — The genome was analyzed to (A) LVP-K1-RBD19 (NP/P) virus in passages 1 and 7 and (B) LVP-K1-RBD19 (P/M) in passages 1 and 7. (DOCX) [file pone.0263684.s006.docx]

S6 Fig.


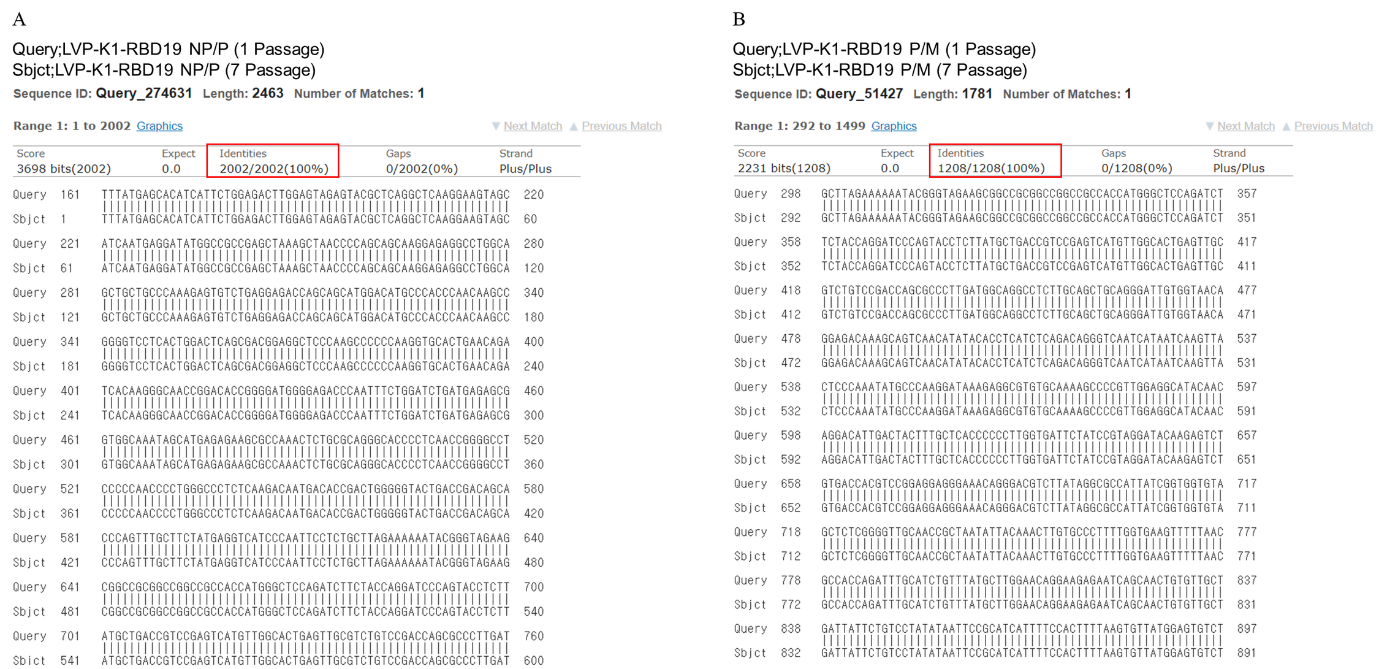


**S6 Fig. Sequence alignment of RBD gene**. The genome was analyzed to (A) LVP-K1-RBD19 (NP/P) virus in passages 1 and 7 and (B) LVP-K1-RBD19 (P/M) in passages 1 and 7.
