# Supplementary material for: The human ACE-2 receptor binding domain of SARS-CoV-2 express on the viral surface of the Newcastle disease virus as a non-replicating viral vector vaccine candidate
Source: PLoS One. 2022 Feb 8;17(2):e0263684. doi: 10.1371/journal.pone.0263684 (PMC8824364; doi:10.1371/journal.pone.0263684)
Supplement: S1 Text — (DOCX) [file pone.0263684.s007.docx]

**S1 text**

Neutralization assay
 A 96-well cell culture dish in which Vero E6 cells were seeded at 1ⅹ10^4^ per well was cultured for 24 hours at 37°C and 5% CO_2_ conditions. After a 1:1 reaction with the isolated antibody and the virus at a concentration of 10^2^TCID_50_/30 ul, it was reacted at room temperature for 30 minutes. After 30 minutes of reaction, 50 ul of the antigen-antibody mixture sample was dispensed on a Vero E6 cell plate washed once with PBS. Incubated for 1 hour at 37°C and 5% CO_2_. After 1 hour of reaction, after removing the existing antigen-antibody mixture sample, 150 ul of Infection Medium was dispensed. Incubated for 72 hours at 37 °C and 5% CO_2_ conditions. The CPE phenomenon was observed through a microscope. After observation, 150 ul of 10% Formalin solution was dispensed per well. After fixing for 30 minutes, all solutions in the well were removed and washed once with PBS. Cells were stained with Crystal Violet staining solution. After 30 minutes of staining, it was washed once with PBS. The median neutralization dose was calculated by the Reed-Munch method.
