# Supplementary material for: The human ACE-2 receptor binding domain of SARS-CoV-2 express on the viral surface of the Newcastle disease virus as a non-replicating viral vector vaccine candidate
Source: PLoS One. 2022 Feb 8;17(2):e0263684. doi: 10.1371/journal.pone.0263684 (PMC8824364; doi:10.1371/journal.pone.0263684)
Supplement: S2 Text — (DOCX) [file pone.0263684.s008.docx]

**S2 text**

Comparison of LVP-K1-RBD19 (NP/P) & LVP-K1-RBD19 (P/M) viruses

1) Construction of RBD expressing cDNA vector between P and M gene

The viral RNA was extracted by RNA preparation kit (Qiagen, hilden, germany Germany) for RNA genome isolation and amplified four fragments by reverse transcription polymerase chain reaction (RT-PCR, Bioneer, Daejeon, South Korea) with the specific 4 pairs primers containing restriction enzyme sites shown in Table 1. RT-PCR was performed by reaction at 42°C for 1 hour and at 94°C for 5 min, followed by a total of 30 cycles of 94°C for 1 min, 60°C for 1 min, and 72°C for 1 min, followed by reaction at 72°C for 7 min. The anti-genomic cDNA construction was sub-cloned to a modified pBR322 low-copy-number plasmid digested Pac I and Pme I restriction enzyme. The pBR322 vector was modified preferably under the control of the T7 RNA polymerase promoter and was positioned so that it was terminated by the hepatitis delta virus (HDV) antigenome ribozyme and T7 terminator gene used to split RNA at the terminus of the NDV genome, thereby allowing viral encapsulation and packaging.

The four cDNA fragments have the same nucleotide sequence at the terminus of 15 bp, and a transgene cassette consisting of a GE-IG-GS sequence and multiple cloning site (MCS) was inserted between the P gene and M gene by overlap cloning to construct the LVP-K1 vector for foreign gene insertion. The gene encoding RBD protein was inserted into the NDV surface expression cassette (genes encoding the F2 subunit including signal sequence and HR4, and F1 subunit including fusion peptide, transmembrane domain and cytoplasmic tail of the NDV fusion protein) for surface expression of NDV to construct a SARS-CoV-2 RBD protein expression gene combination. The SARS-CoV-2 RBD protein expression gene combination was designed and synthesized to have a FseI restriction enzyme recognition site and a kozak sequence at the N terminus, and a FseI restriction enzyme recognition site at the C terminus and to be inserted between the P gene and the M gene of NDV. The LVP-K1 vector for foreign gene insertion and the RBD expression cassette in a ratio of 1 : 3, ligation was performed overnight at 4°C using T4 ligase, and the transformation was performed with *E. coli* TOP10 competent cells using a heat shock method (heat shock). Then, the seeds were determined through colony PCR using primers, forward primer 5’-GGAACAGGAAGAGAATCAGCAAC-3’ and reverse primer 5’- CAACACCATTAGTGGGTTGG -3’. The plasmid midi preparation was performed to obtain a plasmid expressing the RBD of the SARS-CoV-2 spike protein on the surface of NDV.

1. Recovery of the recombinant virus

NDV transcription complex genes, NP, P, and L were separately cloned to pBR322 vectors and used for helper plasmid (pBR322-NP, pBR322-P, pBR322-L). Before one day of the transfection, HEp-2 cells (5ⅹ10^5^ cells/well) were seeded in 6 well plates. Then, the modified vaccinia virus (MVA-T7) was infected with 1 MOI (multiplicity of infection). 2.5 μg, 1.5 μg, 0.5 μg, and 5 μg of pBR322-NP, pBR322-P, and pBR322-L Helper plasmids expressing proteins by the T7 promoter and LVP-K1-RBD19 (P/M), a plasmid expressing RBD of SARS-CoV-2 spike protein on the surface were mixed with Lipofectamine 3000 (Invitrogen, Carlsbad, CA, USA) at an appropriate ratio in the cell line to perform their transfection. The HEp-2 cell supernatant was harvested after incubation at 37°C and 5% CO_2_ for 3 to 4 days. Then, they were inoculated into the allantoic cavity of 9 to 11 days old SPF embryonated egg (Orient Bio, Seongnam, South Korea). The allantoic fluid was collected at 4 days after inoculation. To remove vaccinia virus, allantoic fluid diluted at 10^-3^ with PBS was inoculated into the allantoic cavity of the 9 to 11 days old SPF embryonated egg. After 4 days of inoculation, the allantoic fluid was harvested.

1. Identification of the recovered viruses

LVP-K1-RBD19 (NP/P) and LVP-K1-RBD19 (P/M) viruses were adapted to Vero 76 cells. The cells were cultured as follows. The cells were cultured using Dulbecco's minimum essential medium (DMEM, Gibco, USA) containing penicillin-streptomycin (Gibco, USA) and 10% FBS for cell culture and cultured using a 175 T flask. When the cells grew to form a monolayer of 70 to 80% or more, subculture was proceeded and maintained. The split ratio of Vero 76 cells may be up to 1: 8 and the seeding density is 1 × 10^4^ cells/ml. To conduct a comparative experiment LVP-K1-RBD19 (NP/P) virus and LVP-K1-RBD19 (P/M) virus was infected to the prepared Vero 76 cells. The culture medium of Vero 76 cells was removed to form a monolayer of 70 to 80% or more in a 75 T flask. After that, 10 ml of serum-free DMEM medium was put into the flask and carefully shaken to wash the cells. After repeating this process 2 or 3 times, the viruses (0.1 MOI) were infected in Vero 76 cells and shaken in a 37°C incubator at intervals of 10 minutes and sensitized for 1 hour. After removing the virus solution, 10 ml of serum-free DMEM medium was put into the flask to remove the remaining virus solution. Then, 20 ml of DMEM medium to which 5% FBS was added was put there. The virus was subjected for titer measurement. To test the growth kinetics, the virus was collected at 12, 24, 36, 48, 60, 72, 84, 96 hours to measure the virus titer by the TCID_50_ measurement method.

The viruses were also inoculated into Vero 76 cells again and this process was repeated up to 10 passages to perform the molecular genetic test. The viral RNA was extracted by RNA preparation kit (Qiagen, Hilden, Germany) for RNA genome isolation and amplified by reverse transcription polymerase chain reaction (RT-PCR, Bioneer, Daejeon, South Korea) to identify the viral genome (Table 3). RT-PCR was performed by reaction at 42 ℃ for 1 hour and at 94 ℃ for 5 min, followed by a total of 30 cycles of 94 ℃ for 1 min, 60 ℃ for 1 min, and 72 ℃ for 1 min, followed by reaction at 72 ℃ for 7 min. Sequence alignment of the RBD gene was identified in both LVP-K1-RBD19 (NP/P) and LVP-K1-RBD19 (P/M) virus in passages 1 and 7.

1. Virus concentration and purification

Vero 76 cells were cultured at 3 ⅹ10^5^ cells/mL in 175 cm^2^ t-flask. On the next day, the recombinant virus was inoculated at 0.05 MOI for 2 days following general virus inoculation method. The virus titer was measured at the 2 days post-inoculation. The viruses were clarified by centrifugation at 5,000 g, at 4 ℃ for 10 min to remove debris and the supernatant was collected. The virus was pelleted by ultracentrifugation (rotor No 9, Ultra 5.0, Hanil, Daejeon, South Korea) at 32,000 rpm for 3 hours at 4 ℃. The supernatants were aspirated off, and the virus pellet was re-suspend in TNE Buffer (10 mM Tris-HCl, 20 mM NaCl, 1 mM EDTA). The concentrated virus was purified at ultracentrifuge in the 10 to 40 % sucrose gradient for 3 hours at 32,000 rpm 4 ℃. The purified virus was received in 1 ml fractions and measured UV absorption at 260 and 280 nm. The fraction was used to virus titration following TCID50 measurement titration method. The purified virus fraction was dialyzed using dialysis tubing cellulose membrane (33 mm, lot 3110, Sigma Aldrich, St. Louis, MO, USA) against PBS buffer (pH 7.4) at 4 ℃ overnight.

1. Identification of the RBD viral surface expression

BCA protein analysis was used to measure the protein concentration. Then, 20 μg of protein was separated through 10% SDS-PAGE and transferred on a PVDF membrane. The membranes were reacted with the SARS-CoV-2 Spike RBD Antibody (R&D Systems, Minneapolis, MN USA) and NDV HN protein Polyclonal Antibody (Bioss Antibodies, Woburn, MA, USA) in blocking buffer at room temperature for 1 hour following general western blotting method, followed by goat anti-rabbit IgG-HRP (Invitrogen). Reactive proteins were detected with ECL kit (Invitrogen) following the manufacturer’s protocol.
