# Supplementary material for: The human ACE-2 receptor binding domain of SARS-CoV-2 express on the viral surface of the Newcastle disease virus as a non-replicating viral vector vaccine candidate
Source: PLoS One. 2022 Feb 8;17(2):e0263684. doi: 10.1371/journal.pone.0263684 (PMC8824364; doi:10.1371/journal.pone.0263684)
Supplement: S3 Text — (DOCX) [file pone.0263684.s009.docx]

Supplementary information is available in this paper.

In this study, we determined that antigen concentration was 10 μg of RBD protein expressed on the surface of LVP-K1-RBD19 for the pseudovirus neutralization assay using an HIV-based system (HIV-2019-nCoV-spike pps-myc-Luc). As supplementary data, a graph of neutralizing antibody of RBD protein 10 μg/dose with alum was presented (S1 Fig). Recently, we analyzed the neutralizing antibody titer using the SARS-CoV-2 S clade strain in the BSL3 laboratory of Chungbuk national university (S2 Fig). hACE2 transgenic mouse 5 groups were randomly divided into five mice each in 5 and 10 μg of RBD protein expressed on the surface of LVP-K1-RBD19, and Negative control and one mouse each in the positive control (RBD and Spike). The positive control group was inoculated with RBD or spike protein three times. The 10 μg of RBD and spike protein were inoculated with the proteins mixed with complete at first immunization, incomplete adjuvant at second in two weeks intervals through intramuscular injection, and 10 μg of RBD without adjuvant at one week post second immunization through tail vein injection. LVP-K1-RBD19 (5 and 10 μg RBD protein expressed on the surface of virus) were inoculated twice without adjuvant in 2-week intervals. Compared with the positive control group (RBD and Spike), 5 and 10 μg of RBD protein expressed on the surface of LVP-K1-RBD19 showed significant neutralizing antibody titers. Therefore, we suggest that LVP-K1-RBD19 was an efficient vaccine candidate.

We constructed two different RBD gene insertion sites of the NDV NP-P and P-M. Because most of the previous studies determined P-M sites for foreign gene insertion, but we did not find critical differences between NP-P and P-M sites. Therefore, we constructed RBD genes inserted NP-P and P-M. After finishing the recovery of the virus, we did a growth kinetics comparison test between two recombinant NDV NP-P and P-M (S3 Fig). Both viruses showed similar virus growth kinetics in Vero 76 cells, and RBD protein expression has similar levels (S4 Fig). Finally, we checked RBD gene stability depending on the passage of both recombinant viruses for 10 passage cultures in Vero 76 cells (S5 Fig). We checked RBD gene stability by RBD gene sequencing (S6 Fig). These results showed no mutation occurred at the RBD gene, which was inserted two different sites of the NDV genome and no mutation occurred depend on the passage number. In addition, we make a foreign gene expression cassette like multi cloning site like plasmid DNA, so we could at least 6 weeks for making vaccine strain against genetically variant SARS-CoV-2 virus. Notwithstanding, we must do test cross-reactivity with genetically different strains such as delta, delta plus. It is a really good idea for our future research.

Materials and methods of additional information can be referred to in S1 and S2 Texts.
