# Supplementary figures and images for: The human ACE-2 receptor binding domain of SARS-CoV-2 express on the viral surface of the Newcastle disease virus as a non-replicating viral vector vaccine candidate
Source: PLoS One. 2022 Feb 8;17(2):e0263684. doi: 10.1371/journal.pone.0263684 (PMC8824364; doi:10.1371/journal.pone.0263684)

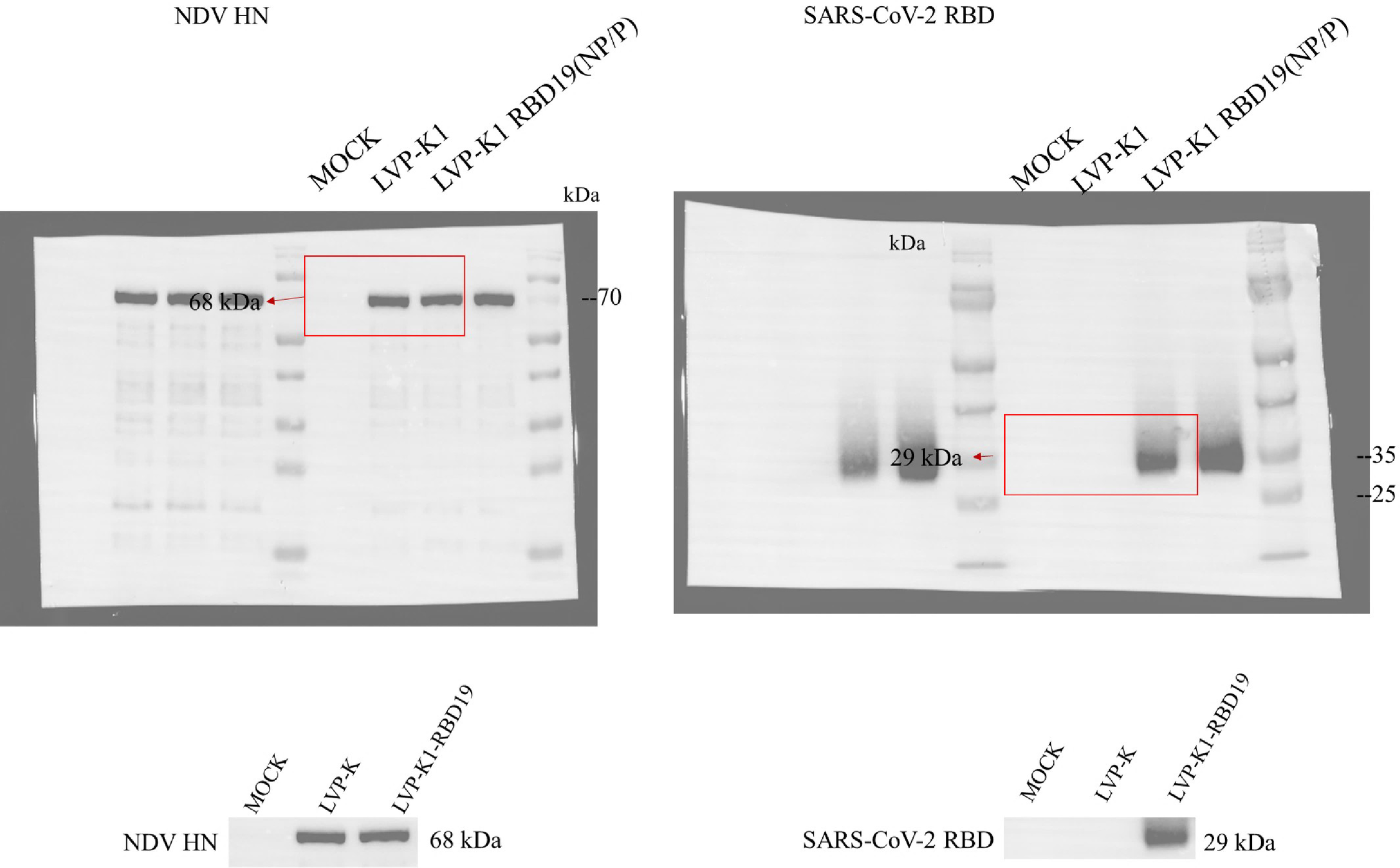

Supplement: S1 Raw images — (TIF) [file pone.0263684.s010.tif]

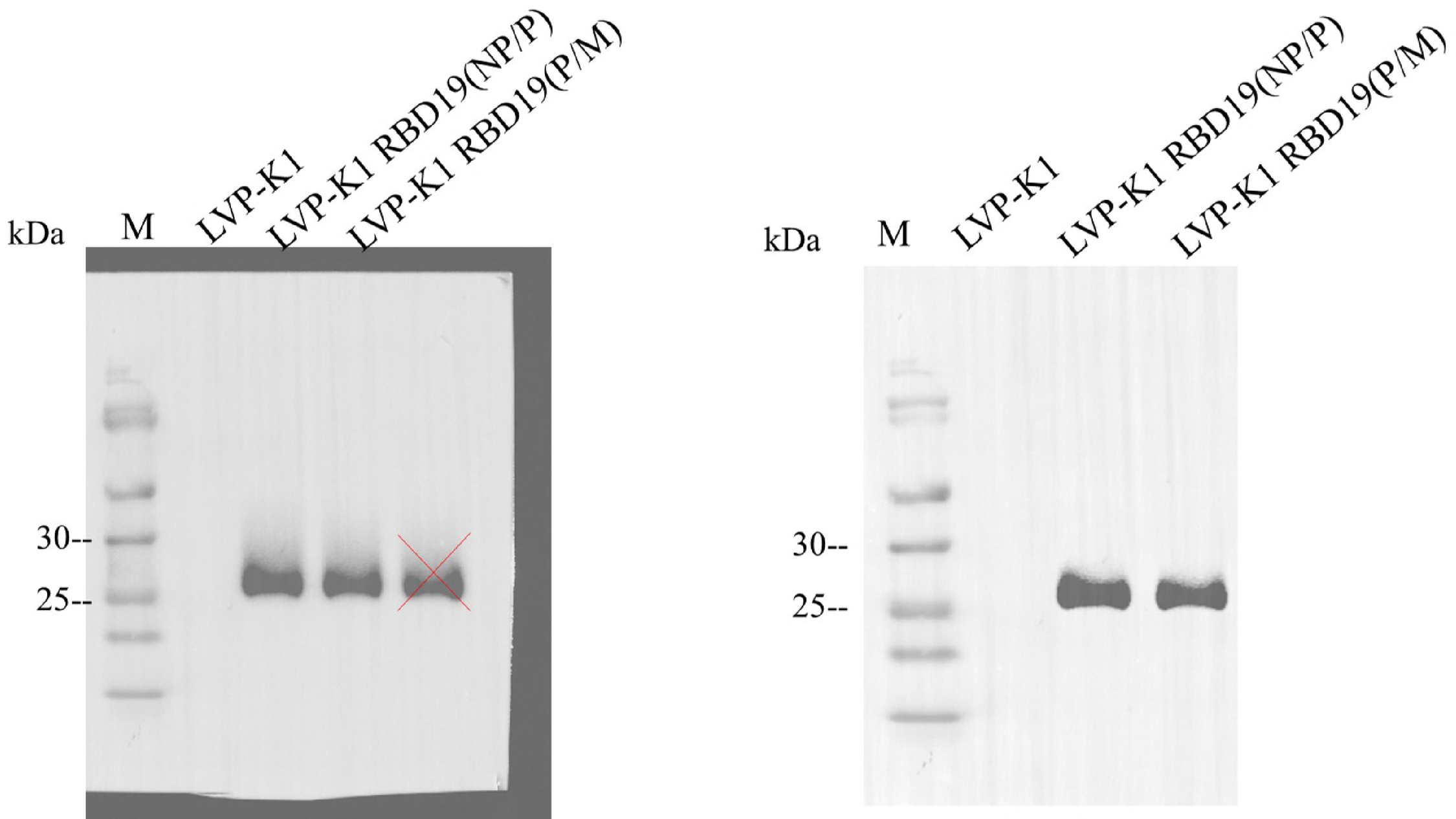

Supplement: S2 Raw images — (TIF) [file pone.0263684.s011.tif]
